# Adequacy of laser diffraction for soil particle size analysis

**DOI:** 10.1371/journal.pone.0176510

**Published:** 2017-05-04

**Authors:** Peter Fisher, Colin Aumann, Kohleth Chia, Nick O'Halloran, Subhash Chandra

**Affiliations:** Agriculture Research & Development Division, Department of Economic Development, Jobs, Transport & Resources, Tatura, Victoria, Australia; Queen's University at Kingston, CANADA

## Abstract

Sedimentation has been a standard methodology for particle size analysis since the early 1900s. In recent years laser diffraction is beginning to replace sedimentation as the prefered technique in some industries, such as marine sediment analysis. However, for the particle size analysis of soils, which have a diverse range of both particle size and shape, laser diffraction still requires evaluation of its reliability. In this study, the sedimentation based sieve plummet balance method and the laser diffraction method were used to measure the particle size distribution of 22 soil samples representing four contrasting Australian Soil Orders. Initially, a precise wet riffling methodology was developed capable of obtaining representative samples within the recommended obscuration range for laser diffraction. It was found that repeatable results were obtained even if measurements were made at the extreme ends of the manufacturer’s recommended obscuration range. Results from statistical analysis suggested that the use of sample pretreatment to remove soil organic carbon (and possible traces of calcium-carbonate content) made minor differences to the laser diffraction particle size distributions compared to no pretreatment. These differences were found to be marginally statistically significant in the Podosol topsoil and Vertosol subsoil. There are well known reasons why sedimentation methods may be considered to ‘overestimate’ plate-like clay particles, while laser diffraction will ‘underestimate’ the proportion of clay particles. In this study we used Lin’s concordance correlation coefficient to determine the equivalence of laser diffraction and sieve plummet balance results. The results suggested that the laser diffraction equivalent thresholds corresponding to the sieve plummet balance cumulative particle sizes of < 2 μm, < 20 μm, and < 200 μm, were < 9 μm, < 26 μm, < 275 μm respectively. The many advantages of laser diffraction for soil particle size analysis, and the empirical results of this study, suggest that deployment of laser diffraction as a standard test procedure can provide reliable results, provided consistent sample preparation is used.

## Introduction

Sedimentation in water has been used as an empirical technique to separate soil into different particle sizes since at least 1708 [1], while the use of Stokes’ Law to calculate sedimentation times for a range of particle sizes is known to have been used as early as 1904 [2]. Two broad techniques for soil particle size analysis (PSA) have been developed using Stokes’s Law. These are the sieve-pipette method that measures a weight concentration and the sieve-hydrometer (or sieve plummet balance) method that measures the suspension density [3]. The differences between these two approaches have been extensively studied (e.g. [3–6]).

Even though the sieve-hydrometer method greatly simplified sedimentation PSA [7], it is still time consuming requiring multiple steps over several days. The laborious nature of sedimentation techniques means that in most studies only a few points on the particle size distribution are measured, usually those corresponding to subjectively derrived thresholds between clay, silt, fine sand, and coarse sand. In comparison PSA using the laser diffraction method (LDM), which was developed in the 1970’s [8], is much faster requiring only minutes to complete a measurement. LDM measurement also has other advantages, particularly the ability to generate the entire particle size distribution (PSD) which is important for: (1) more completely quantifying differences between soils, (2) developing mathematical PSD functions [9], and (3) transferring data between the different particle size classification systems. In a comparison of different measurement techniques [4, 10], LDM instruments had the highest precision (defined as the median of the normalized difference score) to effectively differentiate between samples of silty sediment. The next most precise techniques, which used settling principles, were over an order of magnitude less precise.

The theory and design of modern LDM equipment can be found in the literature (e.g. [11–13]). There are several factors discussed in the literature that can affect the PSD obtained through LDM, which include: the number of detectors in different manufacturers’ designs (e.g. [4, 14]), the choice of measurement time (e.g. [15]), and one of the most important controllable factors affecting measurement results, how the soil sample is prepared (e.g. [12, 15–17]). There are also post-processing factors that can change the LDM PSD, such as the choice of Fraunhofer or Mie diffraction models or the optical parameters used (e.g. [15, 16, 18, 19]), these can, however, be changed and reapplied to the original measurement data.

High reproducibility has been reported from repeated LDM measurement on the same sample of fine-grained sediments [20]. However, the LDM technique uses only approximately one hundredth of the sample mass (usually 0.3–0.5 g) compared to sedimentation techniques (usually 10–40 g). This can be an advantage when the amount of available sample is limited, such as in rhizosphere studies. However, developing a reliable subsampling technique is critical to obtain small samples that are representative of the bulk soil. If subsampling is not done well results can vary widely, for example, Miller and Schaetzl [21] found that 11.5% of 1485 samples used for LDM analysis changed texture class between the first and second soil subsample.

There is no single “correct” way to represent the size distribution of irregular shaped 3-dimensional particles [22, 23]. Although LDM PSA has been accepted as standard practice in some disciplines, such as sediment analysis (e.g. [24]), according to Buurman *et al*. [14] soil scientists have been slow to ‘trade in the labor-intensive’ sedimentation techniques for three reasons: i) cost, ii) sedimentation is the accepted norm, and iii) the degree of correspondence with sedimentation techniques usually deviates from 1:1 and is therefore considered unacceptable. Nearly 20 years on, LDM machines are now becoming far more common and are seen as more cost effective when the labour costs required for sedimentation measurement are considered. LDM, however, is still not seen as the accepted norm for soil analysis, probably due to the deviation in sedimentation and LDM results for the same soil.

The difference between the results of sedimentation methods and LDM is a consequence of the different soil physical properties measured as a surrogate for particle size. For example, there is considerable analysis demonstrating that sedimentation measurements ‘overestimate’ plate-like clay particles due to the inaccurate application of Stokes’ Law [14, 24–28]. Despite the difference in properties measured it is desirable to have a suitable mathematical relationships for inter-conversion between the two techniques [17].

In this paper we report a soil preparation process that overcomes the difficulty of obtaining small, but representative, soil samples essential for accurate LDM measurement. Soil particle size estimation, using this LDM sample preparation process and traditional sedimentation sample preparation, has been undertaken on 22 soil samples collected from four Australian soil Orders. Using these data, this study aimed to address the following objectives:

- Determine whether pretreatment to remove soil carbon makes a difference in reliably estimating particle size distribution using LDM.
- Determine the equivalent LDM cumulative particle sizes corresponding to the SPM cumulative particle sizes of < 2 μm, < 20 μm, and < 200 μm.

## Material and methods

### Soil sampling and preparation

A total of 22 soil samples were collected from four fields in south-western Victoria, Australia. The sites were selected to cover a wide range of soil textures, typical of the agricultural land use in Victoria, to provide a robust comparison of the two techniques. Each field represented a typical example of one of four different major soil Orders: Podosol (B Horizon dominated by the accumulation of compounds of organic matter, aluminium and/or iron); Dermosol (lacking texture-contrast between A and B horizons, with a structured B horizon); Chromosol (strong texture-contrast between A and B horizons with pH>5.5 in the B horizon); and Vertosol (clay content >35%, displaying shrink/swell properties and slickensides and/or lenticular structural aggregates at depth) [29]. Within each field there were three representative sampling sites at approximately 100 m spacing. At each site a soil sample was collected from two depths (0–10 cm and 20–30 cm), referred to here as topsoil and subsoil. Two topsoil samples were omitted due to insufficient soil mass for testing, making a total of 22 rather than 24 samples. The samples were air-dried at 40°C for 48 hours before being sieved to < 2 mm using a brush mill. Soil organic carbon (SOC) content was determined using the Walkley—Black procedure [30]. Electrical conductivity (EC), pH in 1:5 CaCl_2_, pH in 1:5 H_2_O, exchangeable cations (Ca, Mg, K, Na), and CEC were determined using standard techniques [31]. Calcium carbonate content was not measured as all soils had a pH(CaCl_2_) of < 5.7 and are generally known in the region to be calcium carbonate free, which was confirmed using a dilute acid fizz test.

After appropriate homogenization, three subsamples from each soil sample were extracted, one of 25 g and two of 4 g each. The 25 g subsample was used for SPM analysis, while the two 4 g subsamples were used for LDM analysis. Two soil preparation methods were used, referred to as ‘pretreatment’ (P) and ‘no-pretreatment’ (NP), which are described below. All SPM subsamples were subjected to P, while one of the LDM subsamples was prepared using P while the other had NP.

Samples were collected from private land with the owner’s permission for the study, and the study did not involve endangered or protected species.

### Pretreatment

For the pretreatment (P), organic matter was removed from the soil following the method of Mikhail and Briner [32], using sodium hypochlorite solution (100 mL 2M HCl to 1 litre of sodium hypochlorite, 12.5%) at a rate of 100 mL for 25 g samples and 20 mL for 4 g samples. These were left to stand for 16 hours before being centrifuged for five minutes at 1500 rpm and the supernatant decanted from the sample. This differs from the International and Australian Standard methodologies which uses hydrogen peroxide [33, 34], however, the use of sodium hypochlorite has been the standard technique used in the Victorian Government laboratory for many years because it is suitable for local soils that can contain high smectite, mica and vermiculite contents, it is safer (e.g. does not require heating), and may result in greater carbon removal [35–37]. Subsequently, carbonates were removed using 2 M hydrochloric acid for 1 hour at a rate of 100 mL for the 25 g samples and 20 mL for the 4 g samples. Any soluble salts were then removed by rinsing with 0.2 M hydrochloric acid at the rate of 200 mL for the 25 g samples and 40 mL for the 4 g samples. The samples were then repeatedly rinsed with deionized (DI) water. Sodium hexametaphosphate (5%) was added as the dispersion agent at the rate of 20 mL for the 25 g samples in 200 mL of DI water and 8 mL Sodium hexametaphosphate (5%) to the 4 g samples in 40 mL of DI water. All samples were then placed in a 30 kHz ultrasonic bath for 30 minutes, then shaken for 15 minutes on a horizontal reciprocal shaker. The 25 g samples were finally topped up with DI water to make 800 mL, and all samples were placed on an end-over-end shaker for 16 hours prior to analysis.

#### No pretreatment

For the no pretreatment (NP) samples, 40 mL of DI water and 8 mL of sodium hexametaphosphate (5%) were added to 4 g of soil. Samples were placed in a 30 kHz ultrasonic bath for 30 minutes, shaken for 15 minutes on a horizontal reciprocal shaker and finally placed on an end-over-end shaker for 16 hours prior to analysis.

#### Sample riffling

**Fig 1:**
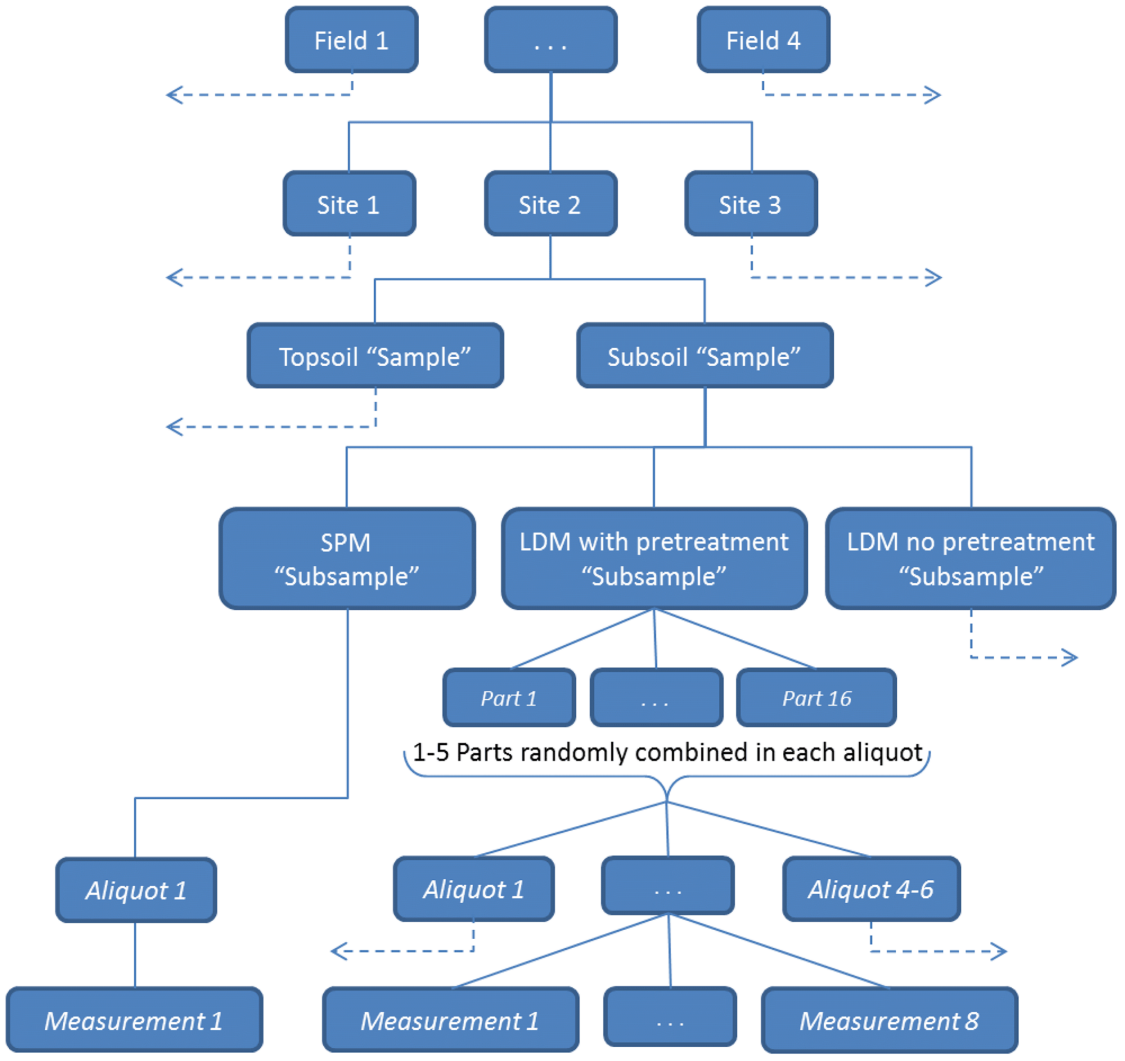
Hierarchical data structure defining the study design. Dashed arrows represent duplicates of the same sampling structure shown. Levels in italic text are not independent replicates. Sieve plummet balance method, SPM; Laser diffraction method, LDM.

Each 25 g subsample, following P, was used as a single aliquot for SPM analysis and measured once. The 4 g subsamples for LDM, following either P or NP, were added to the Hydro 2000G (Malvern Instruments, Malvern, UK) ‘dispersion unit’ which contained approximately 800 mL of DI water. This sample mixture was briefly pumped through the LDM sample cell at maximum pump and stirrer settings. The output tube from the LDM sample cell was connected back to the dispersion unit via a 3-way valve. In this way the flow could be momentarily diverted to riffle the mixture into 16 separate parts, each approximately 30 mL. For LDM measurement, 1 to 5 of these parts were randomly selected and added together to make a sample aliquot (the actual amount of soil used for measurement). In this way four to six aliquots were constructed and each was measured by LDM 6 to 8 times. The hierarchical structure that constituted the research design of this study is illustrated in Fig 1.

### Sieve plummet balance particle size analysis

The 800 mL pretreated suspension was further diluted to 1250 mL using DI water before measurement. A calibrated plummet balance similar to that described by Marshall [38] and mode of operation described by Mcintyre and Loveday [39] and Hutton [40] was used to measure the < 20 μm fraction (at 12 minutes) and the < 2 μm fraction (at 20 hours). Measurements were made in a constant temperature room at 20°C (± 2°) and results were corrected for the temperature effects on density and viscosity of the suspension [39].

Once the clay and silt fractions were determined, the sand (20–2000 μm) was extracted using the decantation technique [41] and dried and sieved (200 μm) into fine and coarse fractions. To enable comparison with LDM particle sizes, the percentages of clay, silt, fine, and coarse sand fractions were corrected to total 100% using the formula Q (% out of 100) = (q*100)/T, where q is observed percent of an individual fraction (clay, silt, fine sand or coarse sand), and T is their total observed percent.

### Laser diffraction particle size analysis

LDM particle size measurements were undertaken in a constant temperature room at 20°C (± 2°) using a Mastersizer 2000 (Malvern Instruments, Malvern, UK) which uses a 52 detector array. The pump speed, stirrer speed, and ultrasonic level were set at 2000 rpm, 800 rpm and 100% respectively. Particle size was calculated on a volume basis using the Mie theory and Malvern proprietary software (version 5.6). Analysis settings used were the General Purpose model with the Irregular Shape Mode. The particle refractive index and particle absorption index used were 1.52 and 0.01 respectively and were the same for both red and blue lasers [21]. The refractive index of the DI water used as the dispersant was 1.33.

When the riffling process was completed the Hydro 2000G (Malvern Instruments, Malvern, UK) ‘dispersion unit’ was cleaned and refilled with approximately 800 mL DI water and 12 mL of sodium hexametaphosphate solution (5%). A 30 second background measurement was made prior to adding soil to account for electrical background and scattering from the optics and ‘clean’ dispersant. Individual sample parts out of the 16 available were then randomly selected and added consecutively to the dispersion unit until the obscuration value was within the manufactures recommended range (10–20%). In this way each aliquot was made up of 1 to 5 parts. Each part was rinsed out of its container using a spray bottle to ensure all the soil was added. When adjustment of the obscuration value was possible, using a different number of parts, to be at either the lower or upper end of the manufactures obscuration range, then 2 of the sample aliquots were tested at each extreme of the range. In this way any effect of the level of obscuration on the measured particle size distribution could be tested. Particle size measurement procedure was initiated immediately following addition of the sample aliquot. Seven—eight repeated measurements were made on each aliquot using a 30 second measurement time [15], which is equivalent to 30,000 individual light scattering measurements. This measurement process was repeated four times for each subsample, each time randomly selecting from the remaining subsample parts.

### Statistical analysis

The statistical treatment of particle size data is complicated by the fact that such data are compositional. That is, they are percentages and sum to 100%. The consequence of this is that an increase in one part will inevitably lead to decreases in other part(s), and the compositions are therefore not independent from each other. Pearson [42] pointed out that because compositions are percentages with a common denominator, they will inevitably exhibit spurious correlations. Several suggestions have been made to address this issue. One is to transform the compositions into radial vectors [43]. The dominant opinion in the field is, however, to transform them into log-ratios [10, 44]. Recent studies have shown that even such transformations only partially solve the problem [45]. In this study we adopted the use of cumulative percentages instead of percentage categories, which also offers only a partial solution, but its virtue lies in its simplicity and interpretability compared to log-ratios or radial vectors.

#### Objective 1. Determination of soil carbon removal effect on LDM measurement

To determine whether removal of soil carbon through pre-treatment affected the reliability of LDM measurements we derived a variant of the non-parametric Kolmogorov-Smirnov (KS) statistic, referred to here as the ${\bar{K}}_{0}$ statistic. We used a permutation test to estimate the p-value of the observed test statistic ${\bar{K}}_{0}$. The smaller this estimated p value is, the stronger the evidence is that the null hypothesis of zero difference between P and NP can be rejected.

To calculate ${\bar{K}}_{0}$, the LDM PSD curves for P and NP were empirically derived at the subsample level, denoted ${\hat{F}}_{NP}$ and ${\hat{F}}_{P}$ respectively, resulting in 22 (P, NP) pairs of LDM curves corresponding to the 22 site by depth combinations. For each of these 22 pairs of curves, we find the particle size *x*^⋆^ on the x-axis corresponding to the maximum absolute difference on the y axis between the two LDM PSD curves ${\hat{F}}_{NP}$ and ${\hat{F}}_{P}$:

$$x^{⋆}≔\underset{x}{\text{argmax}}|{\hat{F}}_{NP}\left(x\right)-{\hat{F}}_{P}\left(x\right)|$$

For the (P, NP) pair i = 1,…,22, we then determine *K*_*i*_ as the signed difference between the cumulative proportions of particle sizes for the ${\hat{F}}_{NP}$ and ${\hat{F}}_{P}$ curves at particle size *x*^⋆^

$$K_{i}={\hat{F}}_{NP}\left(x^{⋆}\right)-{\hat{F}}_{P}\left(x^{⋆}\right)$$

Our test statistics, ${\bar{K}}_{0}$, is then the average of the 22 *K*_*i*_ values

$${\bar{K}}_{0}\;=\frac{1}{22}\overset{22}{\underset{i=1}{\sum }}K_{i}$$

A permutation test [46] is a statistical significance test in which the distribution of the test statistic under the null hypothesis of no difference is obtained by calculating all possible values of the test statistic under rearrangements of the labels on the observed data points. To achieve this we:

1. For *j* = 1,2,…,5000,
  - Randomly (with probability 0.5) swap or not swap the pretreatment labels of each of the 22 pairs of P and NP LDM curves. The swapping is done within each individual (P, NP) pair in order to control for the effects of site and depth. This does not change the magnitude of *K*_*i*_ but randomly either switches or does not switch the sign of *K*_*i*_.
  - Compute the test statistics ${\bar{K}}_{j}$ on this new sample of 22 values.
2. At the end of step 1, we have N = 5000 ${\bar{K}}_{j}$s. The p-value for ${\bar{K}}_{0}$ was estimated as $\#\{\left|{\bar{K}}_{j}\right|\geq \left|{\bar{K}}_{0}\right|\}/N$, which is the proportion of ${\bar{K}}_{j}$s (*j* = 1,…,5000) that are more extreme than or equal to ${\bar{K}}_{0}$.

If the null hypothesis of no pre-treatment effect was rejected a linear mixed effects model, using residual maximum likelihood (ReML) in GenStat [47], was fitted to determine which factors (soil type, depth, and their interaction) were significant contributors to the variation in observed maximum signed differences *K* (the *K*_*i*_ defined earlier) between the cumulative proportions of particle sizes for the NP and the P curves. A linear mixed effects model is a statistical model that contains both fixed effects and random effects. Fixed effects (in our case soil type and soil depth) have levels that are of primary interest and would be used again if this study were repeated. Random effects (in our case sites and samples within sites) have levels that are not of primary interest, but are thought of as a random selection from a much larger set of levels. The two random effects—sites and samples within sites—together define the study design for mixed model analysis of *K*. Accounting for the study design in data analysis using a linear mixed effects model provides more realistic estimates of fixed effect terms [48]. The linear mixed effects model fitted was:

$$K_{shk}=\mu +S_{s}+D_{k}+{\left(S:D\right)}_{sk}+H_{h}+\epsilon_{hk}$$

where *K*_*shk*_ is the maximum signed difference *K* for soil type *s*, depth *k* in site *h*, *μ* is the general mean, *S*_*s*_ is the fixed effect of the *s*^th^ soil type, *D*_*k*_ is the fixed effect of the *k*^th^ depth, (*S*: *D*)_*sk*_ is the fixed effect corresponding to soil type x depth interaction, *H*_*h*_ is the normally distributed random effect for the *h*^th^ site, and *ϵ*_*hk*_ is the normally distributed random residual, both with zero mean and constant variance σ^2^_H_ and σ^2^_ε_ respectively. The ReML-based F statistic was used to determine the statistical significance of the fixed effects.

A linear correlation analysis, based on Pearson’s product moment correlation r, was conducted to identify soil features significantly correlated with *K*. Statistical significance of each correlation was measured using a t test based p value, with t defined as t = r√((n-2)/(1-r^2^)) which has (n-2) degrees of freedom, where n (= 22) is the number of observations [49].

#### Objective 2. Determination of equivalent LDM particle sizes

The LDM curves were averaged at the subsample level (Fig 1) so that both LDM and SPM were measuring distributions at the same level. In other words, for each site by depth combination, we have three SPM measurement classes (at < 2, < 20, < 200μm) and two LDM curves (one each for P and NP). For each of the three SPM measurement classes, we computed the Lin’s concordance correlation coefficient (CCC) [50, 51] between the SPM cumulative particle size value and a range of values from the LDM curve. The CCC measures agreement between two methods by measuring the variation of their linear relationship from the 45° line through the origin. The Pearson correlation coefficient provides a measure that describes the extent to which the points conform to the best fitting line. Lin’s CCC modifies the Pearson correlation coefficient by assessing not only how close the data are about the line of best fit but also how far that line is from the 45° line through the origin, this 45° line representing perfect agreement. The main advantage of CCC is that it is assumption free while other methods like intra-class correlation (ICC), t-test, depends on several assumptions.

## Results

### Sieve plummet balance results and soil chemical properties

The chemical properties of each soil sample and the cumulative percentage of particles < 2, < 20, and < 200 μm measured using SPM are shown in Table 1.

**Table 1:** Chemical properties for each soil sample and the sieve plummet balance method (SPM).

Sample ID	Depth	SOC (%)	EC (ds m^-1^)	pH CaCl_2_	pH water	Chemical properties (meq 100g^-1^)					SPM cumulative % for each particle threshold			
						Ca	Mg	K	Na	CEC	< 2 μm	< 20 μm	< 200 μm	Σ
Podosol														
-	Topsoil	-	-	-	-	-	-	-	-	-	-	-	-	-
1	Subsoil	0.9	0.0	4.1	5.0	0.4	0.4	0.2	0.1	1.1	5.2	11.4	71.1	98.6
2	Topsoil	9.0	0.3	3.9	4.7	54.7	2.5	26.4	6.4	1.5	22.4	27.6	83.3	105.7
3	Subsoil	2.0	0.1	3.9	4.8	0.8	0.8	0.3	0.2	2.1	11.8	19.8	79.0	98.4
4	Topsoil	7.1	0.4	3.7	4.3	159.2	1.5	35.6	5.5	4.1	16.9	23.3	90.7	97.3
5	Subsoil	3.5	0.3	3.9	4.5	248.2	0.9	44.3	7.0	8.5	11.3	16.7	91.6	96.9
Dermosol														
6	Topsoil	4.4	0.2	5.5	6.0	9.6	2.7	1.0	0.2	13.4	24.6	51.3	93.6	93.5
7	Subsoil	1.4	0.1	4.5	5.3	4.0	2.0	0.7	0.1	6.7	27.7	53.3	98.3	99.5
8	Topsoil	4.9	0.1	5.7	6.2	11.6	2.8	0.5	0.1	15.0	22.4	54.5	99.1	93.6
9	Subsoil	1.3	0.1	5.0	5.7	4.7	2.2	0.3	0.2	7.4	23.5	54.9	98.0	102.1
10	Topsoil	4.4	0.3	5.1	5.6	2.7	2.2	29.9	2.4	1.2	28.7	65.3	98.6	94.2
11	Subsoil	1.7	0.3	4.6	5.1	21.0	1.7	35.1	2.1	3.7	27.0	61.5	97.0	100.0
Chromosol														
-	Topsoil	-	-	-	-	-	-	-	-	-	-	-	-	-
12	Subsoil	1.0	0.0	5.6	6.6	3.9	1.0	0.2	0.2	5.2	8.9	36.7	91.9	102.5
13	Topsoil	3.9	0.1	4.5	5.1	6.0	1.2	0.2	0.2	7.6	19.6	46.7	95.8	96.8
14	Subsoil	1.3	0	4.8	5.8	4.4	1.3	0.1	0.1	5.9	19.1	41.7	84.3	104.5
15	Topsoil	5.1	0.2	4.6	5.5	4.3	3.3	0.3	0.8	8.7	17.0	40.0	95.7	94.4
16	Subsoil	1.5	0.1	4.6	5.7	1.8	1.6	0.1	0.3	3.7	13.9	34.9	89.4	102.4
Vertosol														
17	Topsoil	7.4	0.5	5.3	6.0	0.9	1.7	32.8	9.9	1.4	38.3	57.4	92.0	92.4
18	Subsoil	4.2	0.3	5.2	6.1	14.8	13.2	3.2	1.2	32.5	49.2	66.4	96.8	99.9
19	Topsoil	8.7	0.3	5.0	5.7	5.6	1.7	33.7	6.7	1.1	40.7	61.6	95.7	89.8
20	Subsoil	3.9	0.2	5.1	6.0	15.5	14.7	2.0	0.8	33.0	58.0	76.6	97.3	95.7
21	Topsoil	7.3	0.2	5.2	5.9	18.4	8.4	1.1	0.5	28.5	39.0	60.5	94.8	93.3
22	Subsoil	4.1	0.1	5.1	6.1	15.2	13.2	0.7	0.6	29.8	39.9	57.8	93.3	97.6

Cumulative percentages for particle sizes: < 2, < 20, and < 200 μm (< 2000 μm is 100% in all cases). -, missing sample; SOC, soil organic carbon; EC, electrical conductivity; CEC, cation exchange capacity; SPM cumulative % are adjusted so that < 2000 μm is 100% in all cases; Σ, total sum of SPM particle size as observed prior to adjustment.

### Laser diffraction obscuration

Any effect that the level of obscuration has on the measured particle size distribution was tested using five samples: Podosol (#3 NP), Dermosol (#6 P, #8 P), and Chromosol (#13 NP, #15 NP). These samples were selected because the number of subsample parts that make up the aliquot could be varied to achieve obscuration values close to the extreme low and high ends of the manufacturer’s recommended range (10–20% obscuration). The obscuration values, cumulative particle size distribution statistics (tenth percentile, D10; median grain size; D50; ninetieth percentile, D90), and the results from Lin’s CCC between each pair of high and low obscuration particle size distributions are shown in Table 2.

**Table 2:** Cumulative particle size distribution parameters for high and low soil concentrations (average of 2 reps).

Sample ID	Soil Type	Low soil concentration				High soil concentration				Lin’s CCC
		LO	D_10_	D_50_	D_90_	HI	D_10_	D_50_	D_90_	
		%	μm			%	μm			
3	Podosol	10.72	10.6	180.8	273.6	16.36	13.3	197.2	334.6	0.998
6	Dermosol	9.40	2.5	36.0	234.4	17.55	2.4	35.9	248.1	0.999
8	Dermosol	9.59	2.3	21.5	127.9	17.66	2.3	26.9	159.4	0.999
13	Chromosol	11.73	4.5	29.4	188.1	20.46	4.4	29.0	164.8	1.000
15	Chromosol	10.82	5.3	38.5	278.0	19.80	5.1	35.2	240.7	0.999

Last column reports the Lin's CCC for each pair of high and low cumulative particle size distributions. LO, low laser obscuration value; HI, high laser obscuration value; D_10_ tenth percentile; D_50_, median grain size; D_90_, ninetieth percentile

### Effect of pretreatment on laser diffraction results

**Fig 2:**
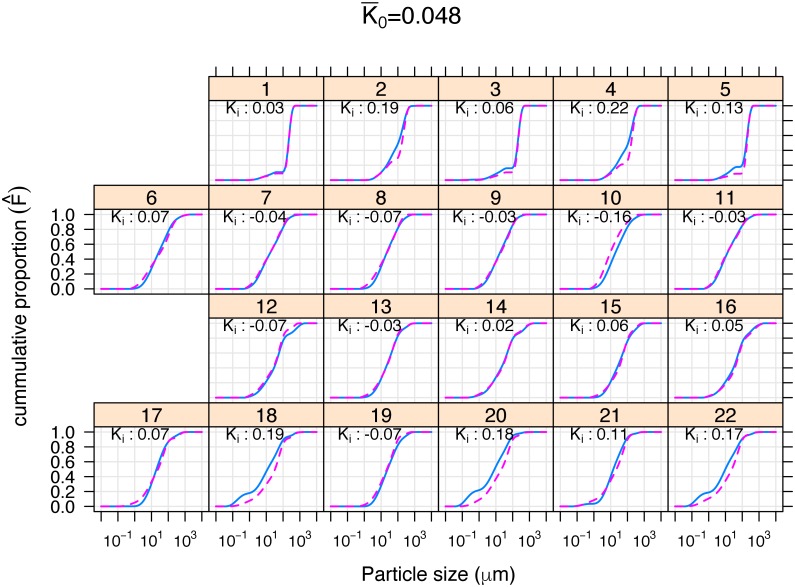
Plot of laser diffraction method cumulative particle size curves with the calculated *K* statistic. Plots are labelled with the sample ID (see Table 1), 1–5, Podosol; 6–11, Dermosol; 12–16, Chromosol; 17–22, Vertosol. Columns are alternating Topsoil/Subsoil. No pretreatment (NP) solid blue line; with pretreatment (P) dashed red line.

The LDM technique provides data for the entire particle size distribution and the average cumulative particle size curves for each site, for P and NP, are shown in Fig 2. The curves generally have a sigmoidal shape using a log-linear scale. The PSD curves for P and NP at each site show, in most cases, considerable similarity (Fig 2). The largest *K*_*i*_ values, and thus the largest differences between the pretreatment and no pretreatment, are found in the Vertosol subsoil (samples #18, 20, 22) and the Podosol topsoil (samples #2, 4). The arithmetic average ${\bar{K}}_{0}$ of these 22 *K*_*i*_ values, which is our test statistic (K_0_), was 0.0481.

The p-value associated with ${\bar{K}}_{0}$, as determined by the permutation test, is 0.022, which leads to rejection of the null hypothesis that pretreatment had no effect using a threshold of p<0.05. Since the null hypothesis of no pre-treatment effect was rejected, the linear mixed effects model was fitted (Table 3). The results indicate a significant effect due to soil type (p = 0.012). We can therefore expect the differences between the pretreatment and no pretreatment ($\bar{K}$) to vary among different soil types. The effect associated with depth (p = 0.540) and for soil type x depth interactions (p = 0.061) were not significant. Results from linear correlation analysis (Table 4) suggest that the only soil feature that bears a statistically significant correlation with the *K* statistic is Na (r = 0.528, p = 0.012). SOC was not found to be significantly correlated with the *K* statistic (r = 0.335, p = 0.128).

**Table 3:** Linear mixed model based estimates of mean *K*_*i*_ for the four soil types, two depths, and their interactions.

Depth	Soil type				Mean
	Chromosol (s.e)	Dermosol (s.e)	Podosol (s.e)	Vertosol (s.e)	
Top soil	0.011 (0.0480)	-0.056 (0.0392)	0.205 (0.0480)	0.039 (0.0392)	0.050 (0.0219)
Subsoil	-0.001 (0.0392)	-0.032 (0.0392)	0.073 (0.0392)	0.182 (0.0392)	0.056 (0.0196)
Mean	0.006 (0.0317)	-0.044 (0.0285)	0.139 (0.0317)	0.110 (0.0285)	

s.e., standard error; p values are: Soil type 0.012, Depth 0.540, Soil type x Depth 0.061 (these F test based p value indicate the statistical significance of overall differences in mean *K* among the four soil types, between the two depths, and among the 12 soil type x depth interactions); LSD 5%: Soil type 0.1054, Depth 0.0676, Soil type x Depth 0.1604 (the LSD can be used to test pairwise differences).

**Table 4:** Correlation of soil features with *K* (n = 22).

	Soil Feature											
	CEC	Ca	EC	K	Mg	Na	SOC	pH_CaCl2_	pH_H2O_	C	C+S	C+S+FS
r	0.307	0.184	0.314	0.318	0.411	0.528	0.335	-0.34	-0.241	0.269	-0.183	-0.236
P	0.165	0.413	0.154	0.149	0.057	0.012	0.128	0.121	0.28	0.227	0.414	0.291

r, correlation coefficient; P, P value; C, clay; S, silt; FS, fine sand

### Laser diffraction equivalents to sand, silt and clay thresholds

**Fig 3:**
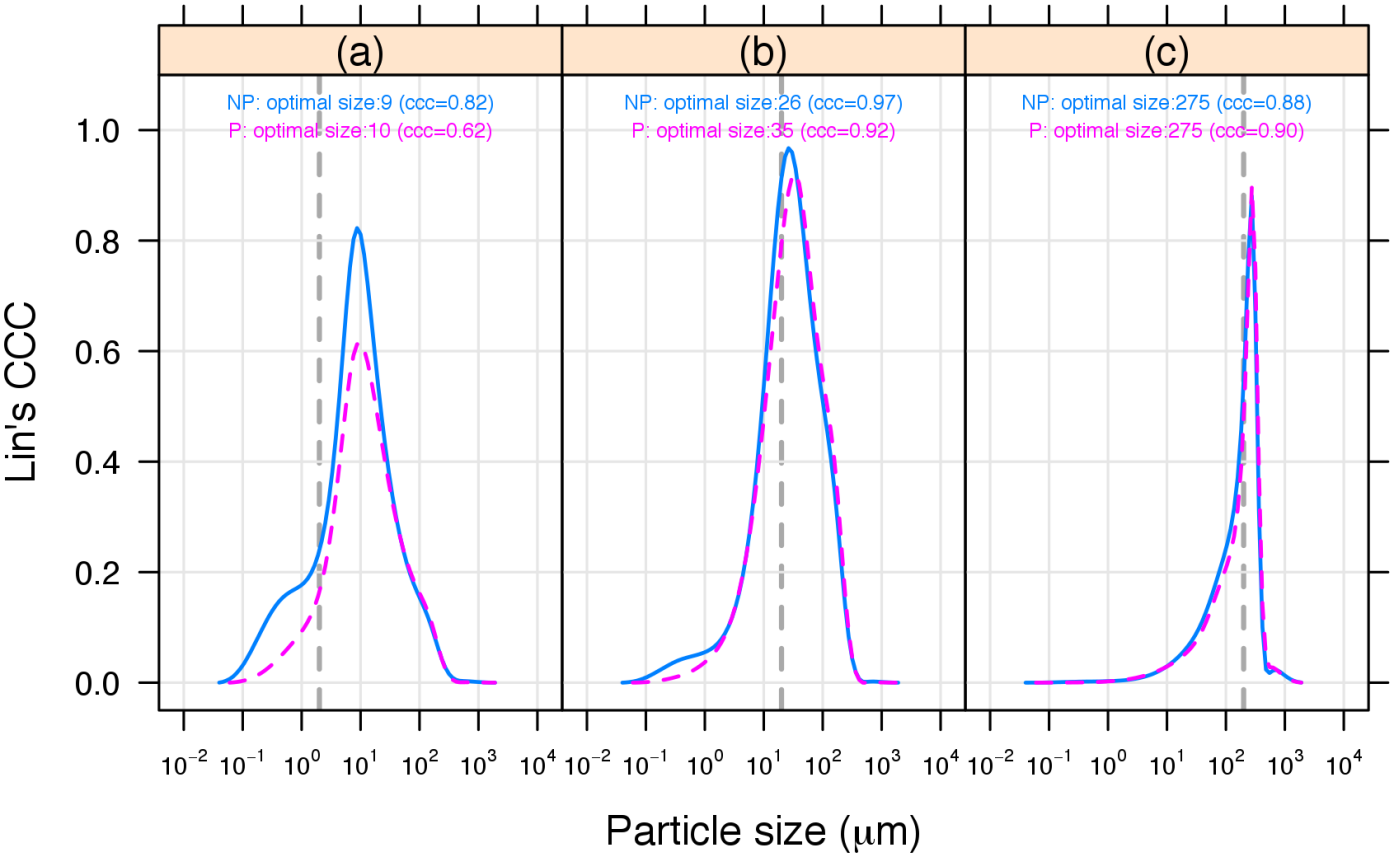
Plots of Lin’s concordance correlation coefficient (CCC) for different laser diffraction (LDM) equivalent sieve sizes. Peak in Lin’s CCC provides the LDM equivalent thresholds that best match the SPM thresholds (shown as a vertical dashed line) of: (a) < 2 μm. (b) < 20 μm. (c) < 200 μm. NP, solid blue line; P, dashed red line.

Fig 3 shows the change in Lin’s concordance correlation coefficient (CCC) for all 22 samples as a function of the LDM cumulative particle sizes above and below each of the SPM particle size thresholds of < 2 μm (Fig 3a), < 20 μm (Fig 3b), and < 200 μm (Fig 3c). The vertical dotted lines in Fig 3 indicate the location of each of these three SPM particle size thresholds. The location of maximum value of Lin’s CCC represents the best LDM equivalent threshold to match each of the three SPM texture thresholds for the 22 samples tested.

**Fig 4:**
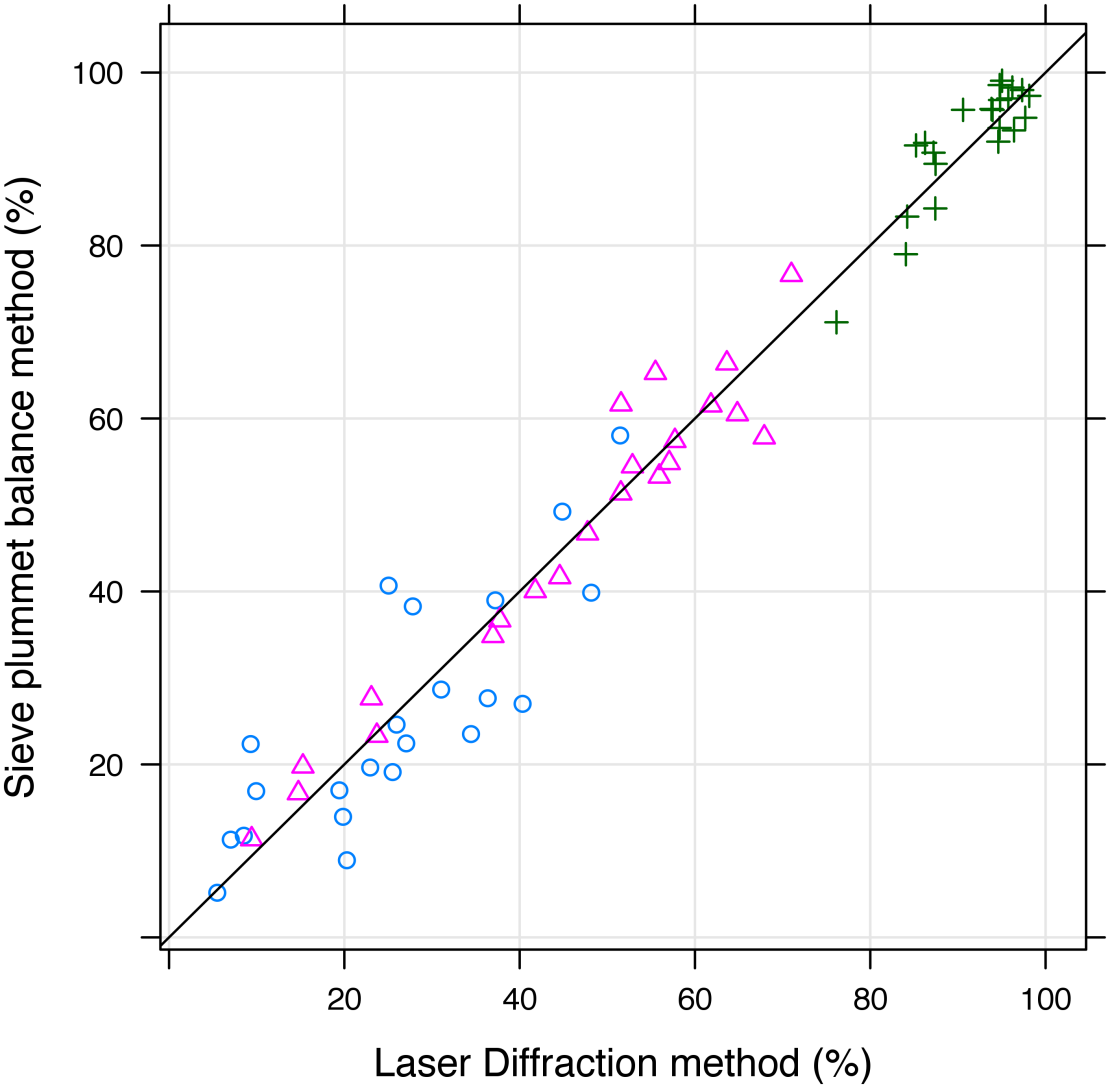
Scatter plot of cumulative particle size (%) using sieve plummet balance (SPM) and laser diffraction (LDM). Results for LDM are NP. Blue circles correspond to coordinates for LDM < 9μm (x-axis), SPM < 2μm (y-axis); red triangles for LDM < 30μm, SPM < 20μm; and green crosses for LDM < 280μm, SPM<200μm.

A scatterplot for all the sample data relating the SPM cumulative particle size percentage values for all 3 SPM thresholds (< 2, < 20, and < 200 μm) with the LDM cumulative particle size percentage values measured at the optimal thresholds of < 9, < 26, and < 275 μm illustrates there is strong agreement with the 1:1 line (Fig 4).

The average and the standard deviation for the LDM (y) data (where y is the estimated LDM thresholds found equivalent to the a standard SPM thresholds of < 2, < 20, < 200 μm) for each of the 22 samples are presented in Table 5. The standard deviation quantifies the reproducibility of the measurements (last two layers in Fig 1).

**Table 5:** Mean and standard deviation (SD) of the empirical distribution of laser diffraction measurements (% less than or equal to thresholds) at 9, 26 and 275 um for non-pretreated (NP) and pretreated (P) samples (n = 24–48).

Sample ID	Non-pretreated samples (NP)						Pretreated samples (P)					
	< 9 μm		< 26 μm		< 275 μm		< 9 μm		< 26 μm		< 275 μm	
	Mean	SD	Mean	SD	Mean	SD	Mean	SD	Mean	SD	Mean	SD
1	5.50	0.32	9.43	0.47	76.15	0.46	5.07	0.43	7.95	0.61	75.00	0.90
2	9.31	1.30	23.08	3.06	84.21	2.49	8.93	0.62	19.42	1.29	77.89	0.80
3	8.53	1.57	15.27	2.09	84.06	6.41	5.31	0.33	9.14	0.52	76.69	0.55
4	9.94	1.31	23.71	2.97	87.22	1.67	8.16	0.86	17.34	1.69	84.07	1.58
5	7.04	0.71	14.76	1.32	85.22	1.26	4.28	0.43	7.72	0.64	85.14	0.99
6	25.96	1.76	51.54	2.09	94.76	2.38	29.18	2.68	44.94	3.89	91.76	4.72
7	36.36	3.80	55.93	5.18	96.21	4.29	37.45	7.92	56.37	12.40	98.03	1.22
8	27.06	2.36	52.87	2.12	95.04	0.79	31.17	2.38	51.71	4.05	98.53	0.96
9	34.45	1.97	57.06	2.53	97.33	1.52	34.34	1.98	54.44	2.89	99.02	0.76
10	31.05	1.91	55.49	2.05	94.76	2.32	47.50	6.30	70.11	9.80	98.59	1.51
11	40.33	2.06	61.83	2.71	95.75	2.70	42.41	2.64	61.00	3.60	97.48	1.87
12	20.29	1.88	37.73	3.30	86.25	6.43	22.60	1.88	39.81	2.56	93.38	2.51
13	22.95	1.00	47.75	1.11	93.83	1.29	24.37	2.19	46.14	4.02	94.21	3.57
14	25.51	1.98	44.57	3.16	87.42	5.54	24.60	3.11	42.57	5.18	88.63	7.52
15	19.43	1.22	41.78	2.05	90.59	2.21	18.78	2.02	35.82	3.65	92.56	2.74
16	19.85	1.96	36.95	3.48	87.45	5.40	17.16	1.73	31.99	2.97	85.19	5.01
17	27.81	6.72	57.71	11.74	94.61	3.76	31.10	3.60	51.16	5.56	92.32	2.96
18	44.87	2.94	63.64	3.14	94.81	2.17	28.10	1.84	44.42	2.73	92.66	3.51
19	25.05	1.38	51.57	1.59	93.95	1.30	29.79	1.77	53.82	2.90	97.09	1.41
20	51.47	3.26	71.00	3.64	98.14	2.02	35.30	2.62	53.49	3.61	95.81	3.27
21	37.22	3.20	64.84	2.98	97.68	1.53	32.93	1.90	53.43	2.83	97.61	1.62
22	48.16	3.96	67.90	4.05	96.40	2.88	32.72	2.07	51.77	2.97	96.83	2.09

## Discussion

### Laser diffraction reproducibility

One source of potential error in the LDM technique is the difficulty to produce a subsample that is representative of the sample and within the manufacturers’ recommended obscuration range (10–20%) when the quantity of soil measured is only 0.3–0.5 g [21]. It is not possible to weigh out subsamples as each soil type has very different obscuration properties. Due to the rapid sedimentation of larger particles it is also not possible to ‘gradually add’ soil from a pre-dispersed subsample. For these reasons, a wet riffling technique was developed. This technique provided considerable flexibility in the quantity of soil in each aliquot while maintaining an accurate subsampling procedure. It was found that the manufacturers recommended obscuration range could be achieved for all the soil Orders tested by varying the number of sample ‘parts’ per aliquot between 1, for the fine textured Vertosol, to 5, for the coarse textured Podosol. Using the Mastersizer pump system for wet riffling is not recommended by the manufacturer to prevent the possibility of equipment damage, however, it was successfully used here in the absence of any specialised wet riffling equipment.

The low standard deviation (SD) compared to the average LDM measurement values (Table 5) suggests high precision for multiple aliquots of the same sample (produced by the proposed wet riffling technique) combined with the repeated LDM measurements on each of the aliquots. A reasonable conclusion from these low SDs is that similar particle size distributions could be expected from even fewer repeated measurements and number of aliquots. Only a single measurement of the SPM technique was made in this study and therefore measurement precision could not be estimated.

It has been suggested by some authors (e.g. [20, 52]) that the obscuration value can have an effect on the LDM particle size distribution, although this has been mainly when obscuration values have been beyond the manufacturers’ recommended range. In our study it was sometimes possible to vary the obscuration value to either the upper or lower end of the manufacturer’s recommended obscuration range by altering the number of parts added to each aliquot. For the soils tested at different obscuration values, Podosol (#3), Dermosol (#6, #8), and Chromosol (#13, #15), there was a strong concordance between low and high obscuration samples in every case with Lin’s CCC > 0.999 (Table 2). This is illustrated by only small differences in the cumulative particle size distribution parameters of D10, D50, D90 with change in obscuration (Table 2). This greatly simplifies LDM sample preparation as no *a priori* knowledge is required about the mass of soil required for testing, provided the measurement is within the recommended manufacturers’ obscuration range.

### Laser diffraction sample pretreatment

To obtain accurate results using SPM it is generally considered necessary to pretreat the soil to remove organic matter and any carbonates. This is a complicated, relatively hazardous, and time consuming process that greatly adds to the measurement cost. To test whether this pretreatment affects the results when using LDM the cumulative particle size curves from all 22 samples were compared with and without pretreatment.

The PSD for each sample for P and NP illustrates that, in most cases, there is very little apparent difference between using or not using pretreatment, whether for topsoil or subsoil (Fig 2). This is supported by the Lin’s concordance correlation coefficient (CCC) analysis for all 22 samples (Fig 3), which demonstrates that LDM using non-pretreated soil produces similar or better agreement (i.e. higher CCC) with the SPM measurements than the pretreated soil, indicating pretreatment in these samples is generally unnecessary. Statistical analysis using the statistic ${\bar{K}}_{0}$ indicated that there was, however, evidence (p = 0.022) for a pretreatment effect. The linear mixed model ‘estimates of mean *K*_*i*_’ for the four soil types, the two depths, and their interactions (Table 3) indicate the greatest difference between the P and NP curves was found for Podosol, followed by Vertosol, while any differences in the Chromosol and Dermosol were not significant. The estimates of mean *K*_*i*_ for the soil type x depth interactions suggest that the major contributors to these interactions, although overall not statistically significant, are Podosol and Vertosol which exhibit large variation between each of the two soil depths in comparison to Chromosol and Dermosol (Table 3).

In all the soils tested carbonate levels were low and therefore any effect of pretreatment can be expected to be due to the reduction in soil organic carbon (SOC), which ranged in this study, prior to any pretreatment, from 0.9% in the Podosol subsoil to 9% in the Podosol topsoil (Table 1). If pretreatment is having an effect on the particle size distribution it would therefore be expected that the magnitude of the effect will be dependent on the quantity of SOC present in the original sample. The linear correlation analysis (Table 4), however, suggested no statistically significant correlation between SOC and the K statistic.

**Fig 5:**
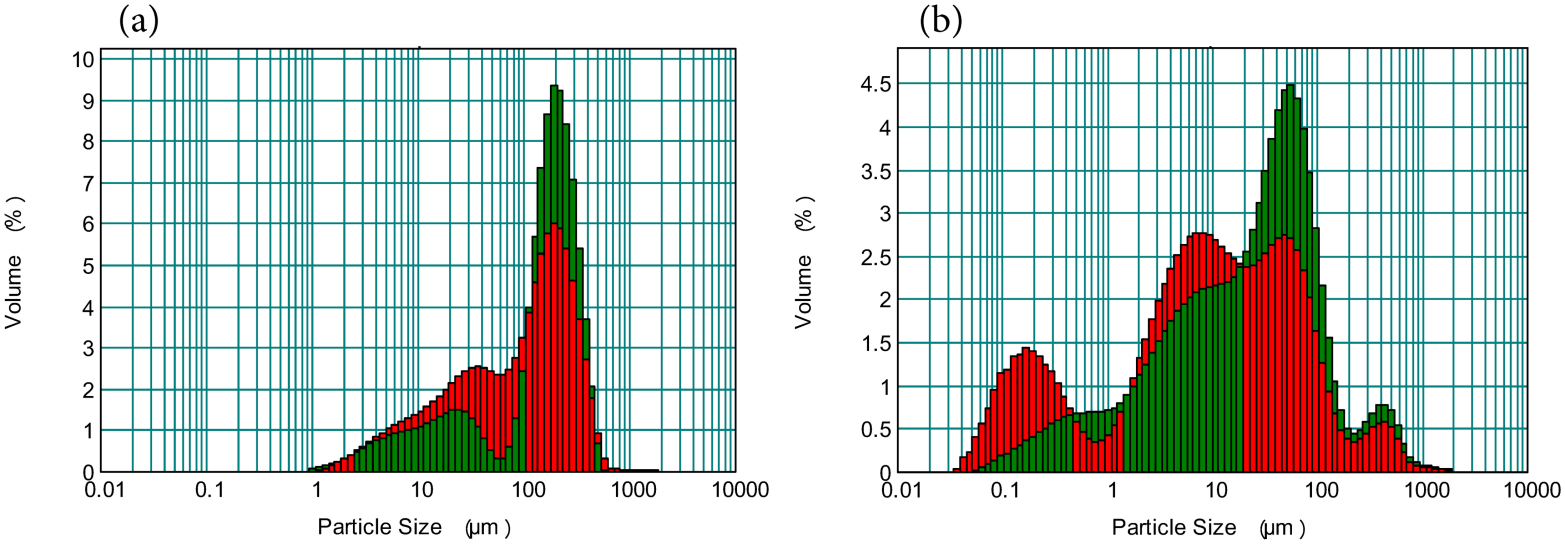
Histogram of particle size distribution. (a) Podosol topsoil. (b) Vertosol subsoil. P, green bars; NP, red bars.

In the Podosol topsoil, P appears to have had the effect of decreasing the proportion of particles in the 30–100 μm range (Fig 5) which has had the effect of producing an unusual flat response in this size range of the cumulative particle size distribution (Fig 2). This is likely to be due to reducing the amount of SOC as these topsoils had very high carbon concentrations, especially for a sandy soil. This size range approximately corresponds to particulate organic carbon which is often defined as greater than 53 μm. Supporting this conclusion is the similar flat response (30–100 μm) found in the Podosol subsoils, which are much lower in SOC (Fig 2).

In the Vertosol subsoil, P resulted in a reduced proportion of particles < 20 μm (particularly < 0.6 μm) resulting in a higher proportion in the 20–200 μm range (Fig 5). However, it is difficult to see how this effect could be attributed to organic carbon removal as there was no similar change due to P in the Vertosol topsoil (Fig 2) which had higher SOC values (Table 1). The carbon removal processes require decanting each sample up to 6 times and it is possible that some clay, especially the very fine particles, is removed during this process. This does not explain, however, why this affect was found in the Vertosol subsoil but not the topsoil.

Although the results from this study suggest that the level of carbon can affect LDM results, this appears to only have a plausible explanation in the sandy Podosol topsoil which is likely to have had a large amount of labile particulate carbon due to years of permanent pasture. Further work is required to validate more broadly whether the removal of soil carbon is justified for LDM particle size analysis and why in some circumstances it makes an apparent difference.

Only a marginal effect of organic matter removal on LDM results was also found by Di Stefano *et al*. [12] for soils with carbon contents ranging from 0.3–7%. Their study, like ours, included end-over-end shaking overnight as part of the soil preparation. In contrast, Vdovic *et al*. [17] found that the common characteristic of samples that showed the greatest shift towards larger particles in the clay/silt range for LDM compared to sedimentation analysis, was a large OM content (> 9%, loss on ignition). They recognised, however, that the mechanical energy from wet-sieving used in the sedimentation pretreatment, but not in the LDM pretreatment, could breakdown aggregation more efficiently. Obtaining a consistent pretreatment method will be critical for the acceptance of LDM for soil particle size analysis. In this paper we have used a high energy approach that included ultrasonic, end-over-end shaking, and the use of a chemical dispersant. This replicates the standard treatment for sedimentation techniques.

### Laser diffraction equivalents to sand, silt and clay thresholds

Gauging the accuracy of the LDM technique is difficult as it requires comparison with a ‘correct’ particle size distribution. All particle size measurement techniques attempt to represent 3-dimensional shapes using a 1-dimensional parameter [23]. The LDM technique uses an equivalent spherical volume biases, whereas the SPM uses an equivalent spherical sedimentation principle. It is therefore not unexpected that the two techniques produce differing proportions of material for a set of arbitrary thresholds, such as < 2 μm, < 20 μm, and < 200 μm. If, however, the different measurement techniques are measuring the relative proportions of particle sizes within a mixture of particle sizes, then accuracy between the techniques can be described by the ability to develop a model that allows data to be converted from one measurement technique to the other.

The LDM technique provides fine resolution data for the entire particle size distribution, and while it is possible to make intermediate measurements, most sedimentation particle size methodologies only measure the quantity of particles corresponding to a small number of categories, e.g. < 2, < 20, < 200 μm, and > 200 μm. The consequence of this is that much of particle size information from the LDM technique had to be discarded when making a direct comparison with the SPM results. It is possible, however, to use the fine resolution of the LDM technique to find the LDM clay-to-silt threshold value, silt-to-fine sand threshold value, and fine sand-to-coarse sand threshold value, that provide the best model prediction of SPM values.

The best agreement (Lin’s CCC close to 1) between the SPM and LDM results, for the 22 samples tested, always occurred for a LDM equivalent value larger than the matching SPM threshold (Fig 3). The strongest agreement for SPM < 2, < 20, and < 200 μm occur at LDM equivalent values of < 9, < 26, and < 275 μm respectively, with corresponding Lin’s CCC values of 0.82, 0.97, and 0.88. The Lin’s CCC values are all higher than 0.80 and, according to Altman (1991) [53], indicate ‘excellent’ agreement between SPM and LDM.

The increase in the threshold between fine and coarse sand is supported by studies comparing LDM measurement of sieve graded sand particles which demonstrated that the mean diameter from LDM was consistently greater than the theoretical sieve class mean [13, 54]. This shift in the fine to coarse sand threshold might not have been identified without the inclusion of the very sandy Podosols in the data set. These soils have a very sharp particle size distribution that tends to occur at the arbitrary 200 μm boundary between fine and coarse sand (Fig 2). Adjusting the LDM fine to coarse sand threshold between 100 to 300 μm can vary the fine sand content in the Podosol subsoil from 20 to 90%. This clearly illustrates the benefits of using a continuous particle size analysis technique, such as LDM, rather than having just three points on a curve without a clear understanding of the shape of the distribution between these points.

The validity of results obtained from sedimentation approaches has been studied elsewhere in the literature. Konert and Vandenberghe [13] and Pabst *et al*. [55] equated the settling velocity of a sphere and a disc using Stokes’ law to show that a sphere of 2 μm diameter has the same settling rate as a disc approximately 8 μm diameter with thickness 0.29 μm when falling broadside. The suitability of this thickness:diameter ratio to represent clay is supported by scanning electron microscope images. Konert and Vandenberghe [13] also supported this theory with measured data that gave a regression slope of 1 when a pipette technique equivalent diameter of < 2 μm was equated with a LDM equivalent diameter of 8 μm. In the theoretical approach of Lu *et al*. [26], a 2 μm Stokes diameter and same disk aspect ratio of 0.037 yields a disk diameter, for random particle orientation, of 8.6 μm, which is very similar to that of Konert and Vandenberghe [13] and to our empirical results. Measurements by Lu *et al*. [26] on kaolinite show that the same cumulative particle size percentage for a 2 μm plummet balance reading was approximately equivalent to a 6.5 μm LDM reading. Kerry *et al*. [18] also found the 8 μm threshold suitable for non-chalk soils, although slightly lower values between 6 and 7.5 μm have also been suggested as the appropriate clay-silt threshold for comparing sedimentation and LDM data [21, 56].

Although a higher threshold value may be useful to translate data between sedimentation and LDM results, it is not necessarily recommended that the threshold values between texture classes should be changed for LDM to those that best match the SPM results [57]. This is because it is most likely that sedimentation techniques are overestimating the clay fraction [13, 22, 26, 55] and should therefore not be used as the reference method [18]. For example, using optical or scanning electron microscopy, numerous particles in the 3–5 μm range and up to 10 μm have been observed in suspensions that have been allowed to settle sufficiently that only particles < 2 μm should be present [13, 25, 55]. The choice of threshold between clay and silt is inevitably somewhat arbitrary and although geologists and soil scientists usually consider the separation to occur at a particle size of 2 μm, sedimentologists often use 4–5 μm, and colloid chemists 1 μm [58].

## Conclusions

The empirical results from this study, using 22 soil samples from four contrasting Australian soil Orders [29], provide strong evidence that laser diffraction (LDM) can be reliably used for routine soil particle size analysis. The use of wet riffling produced highly reproducible results from repeated LDM measurements on the same sample, demonstrating the good precision that can be obtained from the use of this technique. Further investigation is required to derive the optimal number of repeated measurements, number of replicates, and the length of measurement time, for development of a standardised test procedure. The LDM results were found not to be sensitive to the mass of soil analysed provided the obscuration level was maintained within the manufacturer’s recommended range. This result greatly simplifies LDM sample preparation. The use of sample pretreatment generally worsened rather than improved the Lin’s CCC and therefore, for the samples tested, there seems little purpose in adopting a carbon removal pretreatment of LDM samples, although there was an observed effect in the Podosol topsoil and Vertosol subsoil that requires further investigation. Further work on the pros and cons of using pretreatment to remove organic carbon and carbonates for a wider range of soil types is also warranted.

It is important in the discussion of LDM versus sedimentation techniques to remember that neither technique is more ‘correct’ or ‘incorrect’ than the other. Differences in measurement results are mainly a reflection of the different physical principles used to describe 3-dimensional shapes using a 1-dimensional parameter. It is, however, possible, as our results suggest, to translate specific physical thresholds in the particle size distribution between the two techniques of LDM and SPM. The LDM equivalent of the SPM particle size thresholds < 2, < 20, and < 200 μm were found to be < 9, < 26, and < 275 μm respectively.

The LDM approach provides many advantages including considerably greater resolution of the particle size distribution. This information is far more likely to increase our knowledge of the behaviour and differences between soils than just knowing the proportions of sand, silt, and clay. Furthermore, LDM provides a less subjective, and safer test procedure. An important step in the general adoption of the LDM technique will be to develop a standardised methodology, particularly sample dispersion. The study presented here provides one possible methodology.

## Supporting information

S1 Dataset Cumulative particle size date for plummet balance and laser diffraction methods. (XLSX)
